# Phase 1 Study of INBRX-105, a TNFRSF9 (4-1BB) and PD-L1 Bispecific Antibody, in Patients with Select Solid Tumors

**DOI:** 10.1158/2767-9764.CRC-25-0577

**Published:** 2026-02-23

**Authors:** Jong Chul Park, David Berz, Manish R. Sharma, Jyoti Malhotra, Anthony W. Tolcher, Ralph J. Hauke, Justin A. Call, John T. Hamm, Rachel E. Sanborn, Naomi B. Haas, Frank Tsai, Doug R. Adkins, D. Ross Camidge, Alexander I. Spira, Lane Senne, James Kalabus, Brianne O’Neill, Heather Kinkead, Josep Garcia, Erminia Massarelli

**Affiliations:** 1 Massachusetts General Hospital, Boston, Massachusetts.; 2 Valkyrie Clinical Trials, Los Angeles, California.; 3 START Center for Cancer Research – Midwest, Grand Rapids, Michigan.; 4 City of Hope, Duarte, California.; 5 NEXT Oncology, San Antonio, Texas.; 6 Nebraska Cancer Specialists, Omaha, Nebraska.; 7 START Mountain Region, West Valley City, Utah.; 8 Norton Cancer Institute, Louisville, Kentucky.; 9 Earle A. Chiles Research Institute, Providence Cancer Institute, Portland, Oregon.; 10 University of Pennsylvania, Philadelphia, Pennsylvania.; 11 HonorHealth, Scottsdale, Arizona.; 12 Robert Ebert and Greg Stubblefield Head and Neck Tumor Center at Washington University School of Medicine, Alvin J. Siteman Cancer Center, Barnes-Jewish Hospital, St. Louis, Missouri.; 13 University of Colorado Cancer Center, Aurora, Colorado.; 14 Virginia Cancer Specialists, Fairfax, Virginia.; 15 Inhibrx Biosciences, Inc., La Jolla, California.

## Abstract

**Purpose:**

INBRX-105, a tetravalent, PD-L1–targeted TNFRSF9 (4-1BB) agonist, demonstrated preclinical antitumor activity. This first-in-human study evaluated INBRX-105 in solid tumors.

**Patients and Methods:**

This open-label, 4-part, phase 1 study evaluated INBRX-105 alone or with pembrolizumab in adults with locally advanced/metastatic, unresectable solid tumors (NCT03809624). INBRX-105 was administered intravenously once every 2 weeks or 4 weeks in parts 1 (single-agent dose escalation; 0.001–3 mg/kg) and 2 (single-agent expansion) or once every 3 weeks in parts 3 (combination dose escalation; INBRX-105 0.03–1.0 mg/kg) and 4 (combination expansion). Part 2 enrolled patients with checkpoint inhibitor–relapsed/refractory (CPI-R/R) tumors. Part 4 enrolled patients with CPI-R/R or CPI-naïve disease. The primary endpoint was safety and tolerability of INBRX-105. Preliminary clinical response was a secondary endpoint.

**Results:**

Of 160 patients assessed, 81 received monotherapy (median age, 65 years; female, 50.6%), and 79 combination therapy (median age, 64 years; female, 38%). The INBRX-105 maximum tolerated dose was 0.3 mg/kg once every 3 weeks. Head and neck squamous cell carcinoma (*n* = 61) and non–small cell lung cancer (*n* = 25) were the most common tumor types. The most common any-grade INBRX-105–related adverse events (AE) were fatigue (monotherapy, 35.8%; combination, 17.7%), increased aspartate aminotransferase (25.9%; 15.2%), and nausea (19.8%; 17.7%). INBRX-105–related hepatic AEs occurred in 41 patients [monotherapy, *n* = 25 (grade ≥3, *n* = 11); combination, *n* = 16 (grade ≥3, *n* = 6)]. In all patients, the objective response rate was 8.8% [monotherapy, 3.7%; combination, 13.9% (CPI-naïve, 30%; CPI-R/R, 8.6%)]; the disease control rate was 43.1%.

**Conclusions:**

The low response rates and hepatic safety signals observed do not support further clinical development of INBRX-105.

**Significance:**

Tumor resistance to CPIs often develops, underscoring an unmet need. This first-in-human phase 1 trial evaluated INBRX-105—a tetravalent, PD-L1–targeted 4-1BB agonist—alone or with pembrolizumab. Unfortunately, clinical development of INBRX-105 was ended because of hepatotoxicity and limited efficacy. Novel treatment combinations with nonredundant, complementary immunotherapies are needed.

## Introduction

Immuno-oncology therapeutics has reshaped the cancer treatment landscape, modulating the immune response by targeting coinhibitory or costimulatory proteins (1–4). Although most approved agents target immune checkpoint coinhibitory proteins (e.g., PD-[L]1 and CTLA-4), resistance to checkpoint inhibitors (CPI) has increased interest in targeting costimulatory receptors to amplify the antitumor immune response. Tumor necrosis factor receptor superfamily member 9 (TNFRSF9), also known as 4-1BB, is a costimulatory receptor (5). The endogenous ligand of 4-1BB, 4-1BBL, is primarily expressed on professional antigen-presenting cells (5). 4-1BB is upregulated transiently on the surface of T cells following T-cell receptor engagement with (tumor-associated) antigen (5). As with other members of the TNFR superfamily, clustering of ligand-bound 4-1BB receptors on the cell surface is critical for robust downstream signaling (6, 7).

Agonistic 4-1BB monoclonal antibodies have shown some antitumor activity in early clinical studies, including in combination with anti–PD-1 therapies (4, 7, 8). However, the hepatotoxicity observed, potentially due to indiscriminate activation of 4-1BB by these agonists, has limited their therapeutic window (7, 9–11). To potentially improve targeting of 4-1BB activation to the tumor microenvironment, bispecific antibodies with a 4-1BB agonist component and a PD-L1 antagonist component—such as acasunlimab (12, 13), ATG-101 (14, 15), and MCLA-145 (16)—are in development.

INBRX-105 is a humanized, tetravalent, PD-L1–targeted 4-1BB agonist composed of two single-domain antibodies that activate 4-1BB without blocking 4-1BBL binding and two that antagonize PD-L1, with an Fc region engineered to disable its effector functions. INBRX-105 was designed to localize 4-1BB agonism to areas of high PD-L1 expression, such as the tumor microenvironment, as this may potentially minimize the systemic toxicity observed with prior 4-1BB agonists. Additionally, localized antagonism of PD-L1 paired with 4-1BB activation may further promote an antitumor immune response.

Preclinically, INBRX-105 blocked binding of PD-1 to PD-L1 (Supplementary Fig. S1A) and induced signaling downstream of 4-1BB in a PD-L1–dependent manner (Supplementary Fig. S1B; ref. 17), with the binding domains of INBRX-105 having greater binding affinity for PD-L1 versus 4-1BB (Supplementary Fig. S1C and S1D). *In vitro*, INBRX-105 resulted in greater T-cell modulation than a constitutive 4-1BB agonist that was provided alone or in combination with a PD-L1 antagonist (Supplementary Fig. S2A and S2B; ref. 17). *In vivo* studies showed that INBRX-105-a, a murine INBRX-105 surrogate, demonstrated potent antitumor activity, including T-cell tumor infiltration and induction of T-cell memory in PD-L1+ mouse tumor models (Supplementary Fig. S3A and S3B; ref. 17). Addition of an orthogonal anti–PD-1 antibody to INBRX-105-a resulted in enhanced antitumor activity in mouse tumor models and improved blockade of PD-1/PD-L1 interactions compared with INBRX-105-a alone, which may have resulted in increased availability of endogenous PD-L1 and thus more targets for INBRX-105 to bind (Supplementary Fig. S4A–S4C; ref. 17). These findings supported the evaluation of INBRX-105 in a clinical trial.

We present findings from the phase 1, first-in-human study of INBRX-105 alone and in combination with pembrolizumab in patients with select solid tumors.

## Patients and Methods

### Study design

This was an open-label, multicenter, 4-part, nonrandomized, phase 1 study conducted between January 31, 2019, and October 21, 2024, to evaluate the safety and preliminary antitumor activity of INBRX-105 in patients with select solid tumors (trial registration ID: NCT03809624).

Part 1 was a single-agent dose escalation that used an accelerated titration (part 1a) and a 3 + 3 algorithm design (part 1b) to evaluate INBRX-105 doses ranging from 0.001 mg/kg to 3 mg/kg (Supplementary Fig. S5). In part 1, the dose-limiting toxicity (DLT) window was the first treatment cycle (28 days). For patients in part 1a whose dose levels were escalated, the DLT window was cycles 1 and 2.

Part 2 (single-agent dose expansion) evaluated five cohorts of patients with various tumor types (see the “Eligibility” section for more information) that were relapsed or refractory to checkpoint inhibitors (CPI-R/R). Patients with CPI-naïve disease could be eligible if CPIs were not the standard of care. A DLT window was not specified for part 2 in the protocol, but toxicities associated with the initial recommended phase 2 dose (RP2D) of 1 mg/kg were designated as DLTs. The dose of INBRX-105 was subsequently reduced to 0.3 mg/kg.

Part 3 (combination therapy dose escalation; modified toxicity probability interval design) evaluated INBRX-105 at doses ranging from 0.03 to 1 mg/kg in combination with pembrolizumab 200 mg, with both agents given on day 1 once every 3 weeks. In part 3, the DLT window was the first treatment cycle (21 days).

Part 4 (combination therapy dose expansion) consisted of five cohorts: three cohorts enrolled patients with CPI-R/R disease and two cohorts enrolled patients with CPI-naïve disease (Supplementary Fig. S5). DLTs were not assessed in part 4.

This study was conducted in compliance with the Declaration of Helsinki, the International Council for Harmonisation Guideline for Good Clinical Practice, and applicable national and local laws and regulatory requirements. The protocol was approved by the independent ethics committee or institutional review board at each site. All patients provided written informed consent before participation.

### Eligibility

Eligible patients were men or women ages ≥18 years with locally advanced or metastatic unresectable solid tumors whose disease had progressed despite standard therapy and for whom no further standard therapy existed or who refused available standard treatment options.

Parts 1 and 3 enrolled patients with any solid tumor type. Parts 2 and 4 enrolled subcohorts of patients with specific tumor types that were either CPI-R/R or CPI-naïve (Supplementary Fig. S5). In part 2, the following tumor types were evaluated: non–small cell lung cancer (NSCLC; cohort C1); cutaneous melanoma (or any solid tumor) amenable to on-treatment tumor biopsies (cohort C2); head and neck squamous cell carcinoma (HNSCC) amenable to on-treatment biopsies (cohorts C3 and C5); and gastric or gastroesophageal junction adenocarcinoma, renal cell carcinoma, or urothelial (transitional) cell carcinoma (cohort C4). Patients were required to have CPI-R/R disease, but patients with CPI-naïve disease could be eligible if CPIs were not the standard of care.

In part 4, the following tumor types were evaluated: CPI-R/R NSCLC (cohort E1); CPI-R/R cutaneous melanoma (cohort E2); CPI-R/R HNSCC or solid tumors with microsatellite instability/tumor mutation burden–high or mismatch repair deficient (cohort E3); CPI-naïve NSCLC (cohort E4); and CPI-naïve HNSCC (cohort E5).

CPIs could be anti–PD-(L)1 or –CTLA-4 monoclonal antibodies for tumor types in which these were approved. Required thresholds for PD-L1 tumor proportion score (TPS) or combined positive score (CPS) by immunohistochemistry (IHC) varied by subcohort (Supplementary Fig. S5). Existing PD-L1 IHC results were accepted if the antibody clones 22C3 or SP263 were used, which have demonstrated a high concordance rate in multiple tumor types, including NSCLC (18) and HNSCC (19); otherwise, archival tissue or a fresh biopsy was centrally tested.

Patients were not eligible if they had grade ≥3 immune-related adverse events (irAE), irAEs that led to discontinuation of prior immunotherapy, or residual irAEs from previous immunomodulatory therapy that had not recovered to grade 1 or better. Prior exposure to 4-1BB agonists was also not permitted.

### Outcomes

The primary endpoint was safety and tolerability of INBRX-105. Treatment-emergent adverse events (TEAE) were recorded by the investigator using the Medical Dictionary for Regulatory Activities preferred terms and graded using the National Cancer Institute Common Terminology Criteria for Adverse Events v5.0. The secondary endpoints were pharmacokinetics (PK) estimated using standard noncompartmental methods, incidence of antidrug antibodies (ADA), and preliminary clinical response. Measures of response included objective response rate [ORR; best overall response of complete response (CR) or partial response (PR)] by Response Evaluation Criteria in Solid Tumors v1.1 and disease control rate (DCR; best overall response of CR, PR, or stable disease).

### Statistical analysis

Combined data (data cutoff: October 21, 2024) from all study parts are reported here by therapy received (INBRX-105 alone or in combination with pembrolizumab). The safety analysis set comprised all patients who received ≥1 dose of INBRX-105. The efficacy analysis set included all patients who met entry criteria for enrollment, regardless of whether they received study treatment. The ORR was determined for the efficacy analysis set and for evaluable patients (i.e., patients with ≥1 postbaseline assessment and measurable target disease at baseline). Descriptive statistics were used for other clinical and PK parameters.

## Results

### Patients

This study enrolled 160 patients with locally advanced or metastatic solid tumors; all received ≥1 dose of INBRX-105 (monotherapy, *n* = 81; combination, *n* = 79). The median age was 65 years for patients who received monotherapy and 64 years for those who received combination therapy (Table 1; Supplementary Table S1). Almost half of the patients (49.4%) who received monotherapy and 62% who received the combination were male. Nearly all patients had stage III or IV cancer at study entry (monotherapy, 97.5%; combination, 94.9%). Several different tumor types were included, but HNSCC (monotherapy, 37%; combination, 39.2%) and NSCLC (12.3%; 19%) were the most common. Most patients had received previous treatment; only five patients had not received ≥1 prior systemic therapy (monotherapy, *n* = 1; combination, *n* = 4). Approximately half of the patients had previously received immunotherapy (monotherapy, 55.6%; combination, 50.6%).

**Table 1.** Patient demographics and baseline characteristics.

Characteristic	INBRX-105 (*n* = 81), *n* (%)	INBRX-105 + pembrolizumab (*n* = 79), *n* (%)
Age, median (range), years	65 (21–80)	64 (37–92)
Sex	​	​
Female/male	41 (50.6)/40 (49.4)	30 (38)/49 (62)
Race^a^	​	​
White/other	60 (74.1)/21 (25.9)	62 (78.5)/19 (24.1)
Cancer stage at study entry	​	​
I/II^b^	1 (1.2)	1 (1.3)
III/IV	79 (97.5)	75 (94.9)
Unknown/missing	1 (1.2)	3 (3.8)
Disease status at study entry	​	​
Metastatic/locally advanced	75 (92.6)/6 (7.4)	76 (96.2)/3 (3.8)
Cancer type	​	​
Head and neck cancer	30 (37)	31 (39.2)
NSCLC	10 (12.3)	15 (19)
Gastric cancer	8 (9.9)	4 (5.1)
Colon cancer	7 (8.6)	7 (8.9)
Pancreatic cancer	7 (8.6)	1 (1.3)
Renal cancer	4 (4.9)	1 (1.3)
Melanoma	1 (1.2)	9 (11.4)
Other^c^	14 (17.3)	11 (13.9)
Prior systemic therapy^d^	​	​
Any	80 (98.8)	75 (94.9)
Chemotherapy	71 (87.7)	63 (79.7)
Immunotherapy^e^	45 (55.6)	40 (50.6)
Molecularly targeted therapy	10 (12.3)	11 (13.9)
Biologics^f^	3 (3.7)	0
Hormone therapy	0	1 (1.3)
Other	15 (18.5)	9 (11.4)
None	1 (1.2)	4 (5.1)

a Patients could make ≥1 selection.

b Patients with early-stage disease and no available treatment options were included.

c Soft tissue sarcoma, *n* = 3; ovarian cancer, leiomyosarcoma, bile duct cancer, breast cancer, bladder cancer, each *n* = 2; non–Hodgkin lymphoma (diffuse large B-cell lymphoma), renal cell cancer, uterine sarcoma, liver cancer, endometrial cancer, fibrosarcoma, carcinoma of unknown primary, appendiceal adenocarcinoma, small intestine cancer, gallbladder cancer, mesothelioma, and conventional chondrosarcoma, each *n* = 1.

d Patients may have received ≥1 type of therapy.

e A total of 27 patients who received prior immunotherapy were categorized incorrectly as having received prior “chemotherapy” or “other” therapy.

f Therapies were categorized incorrectly by the site and included bevacizumab, anlotinib, and an investigational antibody–drug conjugate.

Overall, 39 patients (48.1%) discontinued monotherapy and 24 (30.4%) discontinued the combination (Supplementary Table S2). Among those who discontinued INBRX-105 monotherapy, AEs were the most common reason reported at the end of treatment [41% (16/39)]. Patient withdrawal and investigator decision were the most common reasons for treatment discontinuation among those who discontinued combination therapy [each, 25% (6/24)]. Patients received a median of 2 cycles of INBRX-105 monotherapy (range, 1–35) or a median of 4 cycles of combination therapy (range, 1–33).

### Safety

TEAEs of any cause were reported in all patients who received INBRX-105 monotherapy (grade ≥3, 75.3%) and in 98.7% of those who received combination therapy (grade ≥3, 63.3%; Table 2; Supplementary Table S3). DLTs, per investigator assessment, occurred in 10 patients (monotherapy, *n* = 8; combination, *n* = 2), with increased liver enzymes (monotherapy, *n* = 5; combination, *n* = 2) being the most common (Table 3). In patients who received monotherapy, most DLTs occurred at the 1- or 3-mg/kg dose levels. After DLTs occurred at 3 mg/kg in part 1, 1 mg/kg was determined to be the maximum tolerated dose (MTD) and RP2D of INBRX-105; however, the dose was subsequently reduced to 0.3 mg/kg after grade 3/4 liver-related AEs occurred in part 2. Both patients with DLTs in the combination subgroup (increased liver enzymes, *n* = 2) had received 1 mg/kg INBRX-105 in part 3. Thus, 0.3 mg/kg was selected as the MTD and RP2D in the combination subgroup and evaluated in part 4.

**Table 2.** Most common TEAEs.

Preferred term	INBRX-105 (*n* = 81), *n* (%)		INBRX-105 + pembrolizumab (*n* = 79), *n* (%)	
	Any grade	Grade ≥3	Any grade	Grade ≥3
Any-cause TEAEs^a^	81 (100)	61 (75.3)	78 (98.7)	50 (63.3)
Fatigue	42 (51.9)	7 (8.6)	29 (36.7)	5 (6.3)
Anemia	27 (33.3)	12 (14.8)	22 (27.8)	6 (7.6)
Nausea	24 (29.6)	1 (1.2)	24 (30.4)	2 (2.5)
AST increased	26 (32.1)	7 (8.6)	19 (24.1)	5 (6.3)
Pyrexia	21 (25.9)	0	21 (26.6)	0
ALT increased	21 (25.9)	7 (8.6)	13 (16.5)	5 (6.3)
Dehydration	17 (21)	1 (1.2)	17 (21.5)	0
Constipation	22 (27.2)	1 (1.2)	9 (11.4)	0
Decreased appetite	20 (24.7)	2 (2.5)	10 (12.7)	2 (2.5)
Diarrhea	16 (19.8)	3 (3.7)	14 (17.7)	0
Arthralgia	17 (21)	1 (1.2)	12 (15.2)	0
Dyspnea	16 (19.8)	6 (7.4)	13 (16.5)	3 (3.8)
Hypokalemia	16 (19.8)	1 (1.2)	11 (13.9)	0
Hyponatremia	18 (22.2)	3 (3.7)	9 (11.4)	1 (1.3)
Headache	12 (14.8)	0	14 (17.7)	0
Vomiting	16 (19.8)	1 (1.2)	9 (11.4)	1 (1.3)
Blood alkaline phosphatase increased	15 (18.5)	5 (6.2)	9 (11.4)	3 (3.8)
Lymphopenia	11 (13.6)	6 (7.4)	13 (16.5)	5 (6.3)
Thrombocytopenia	13 (16)	3 (3.7)	11 (13.9)	4 (5.1)
INBRX-105–related TEAEs^b^	72 (88.9)	30 (37)	57 (72.2)	23 (29.1)
Fatigue	29 (35.8)	5 (6.2)	14 (17.7)	1 (1.3)
AST increased	21 (25.9)	7 (8.6)	12 (15.2)	4 (5.1)
Nausea	16 (19.8)	0	14 (17.7)	0
Pyrexia	14 (17.3)	0	14 (17.7)	0
ALT increased	17 (21)	7 (8.6)	10 (12.7)	5 (6.3)
Anemia	15 (18.5)	3 (3.7)	5 (6.3)	1 (1.3)
Thrombocytopenia	9 (11.1)	3 (3.7)	10 (12.7)	3 (3.8)
Chills	10 (12.3)	0	7 (8.9)	0
CRS	6 (7.4)	1 (1.2)	11 (13.9)	1 (1.3)
Diarrhea	8 (9.9)	2 (2.5)	9 (11.4)	0
Arthralgia	10 (12.3)	0	6 (7.6)	0

a Shown are any-cause TEAEs reported in ≥15% of all patients (*N* = 160).

b Shown are INBRX-105–related TEAEs reported in ≥10% of all patients (*N* = 160). Related TEAEs were those with a relationship of possible, probable, or very likely/certainly related to INBRX-105 as determined by the investigator.

**Table 3.** DLTs.

DLT preferred term, *n* (%)	INBRX-105 monotherapy^a^						INBRX-105 + pembrolizumab^a^	
	0.003 mg/kg (*n* = 1)	0.1 mg/kg (*n* = 6)	0.3 mg/kg (*n* = 20)^b^	1 mg/kg (*n* = 12)^c^	3 mg/kg (*n* = 3)	Total (*n* = 81)	1 mg/kg (*n* = 8)	Total (*n* = 30)^d^
Patients with ≥1 DLT^e^	1 (100)	1 (16.7)	1 (5)	3 (25)	2 (66.7)	8 (9.9)	2 (25)	2 (6.7)
AST increased	0	0	1 (5)	1 (8.3)	1 (33.3)	3 (3.7)	1 (12.5)	1 (3.3)
ALT increased	0	0	0	1 (8.3)	1 (33.3)	2 (2.5)	0	0
Immune-mediated hepatitis	0	1 (16.7)	0	1 (8.3)	0	2 (2.5)	0	0
Neutrophil count decreased	1 (100)	0	0	0	0	1 (1.2)	0	0
Arthritis	0	0	0	1 (8.3)	0	1 (1.2)	0	0
Blood ALP increased	0	0	0	0	1 (33.3)	1 (1.2)	0	0
Myalgia	0	0	0	0	1 (33.3)	1 (1.2)	0	0
Transaminases increased	0	0	0	0	0	0	1 (12.5)	1 (3.3)

DLT preferred terms are listed as reported by study sites.

Abbreviations: ALP, alkaline phosphatase.

a The table includes only individual dose levels at which ≥1 DLT occurred; however, the total columns include all patients in the Safety Analysis Set (i.e., all dose levels combined). Therefore, the sum of the dose groups may not equal the total columns. All patients who experienced a DLT while receiving single-agent therapy received INBRX-105 every 2 weeks. Both patients who experienced a DLT while receiving combination therapy received INBRX-105 and pembrolizumab every 4 weeks.

b The 1 mg/kg dose level of single-agent therapy included patients from both parts 1 and 2, including 3 patients from part 1 (cohort A6) and 17 patients from part 2 [cohorts C1 (*n* = 7), C3 (*n* = 5), and C4 (*n* = 5)].

c For the single-agent therapy group included in this table, the 3 mg/kg dose level included patients from both parts 1 and 2, including six patients from part 1 (cohort B1) and six patients from part 2 [cohorts C1 (*n* = 2), C3 (*n* = 3), and C4 (*n* = 1)].

d These patients were in part 3 only; DLTs were not evaluated in part 4.

e Patients may have experienced multiple DLT preferred terms. These patients were included in the counts for each applicable DLT preferred term but are counted only once in the row of patients with ≥1 DLT.

INBRX-105–related TEAEs were reported in 88.9% and 72.2% of patients who received monotherapy or combination, respectively. The most common INBRX-105–related TEAEs were fatigue (monotherapy, 35.8%; combination, 17.7%), increased aspartate aminotransferase (AST; 25.9%; 15.2%), increased alanine aminotransferase (ALT; 21%; 12.7%), nausea (19.8%; 17.7%), anemia (18.5%; 6.3%), and pyrexia (17.3%; 17.7%). Cytokine release syndrome (CRS) as reported by the investigator occurred in 17 patients (monotherapy, *n* = 6; combination, *n* = 11); most cases were grade 1 or 2. INBRX-105–related hepatic TEAEs were reported in 41 patients (monotherapy, *n* = 25; combination, *n* = 16) and were grade ≥3 in 10.6% of patients overall (*n* = 11; *n* = 6; Supplementary Table S4). Most hepatic TEAEs were reversed rapidly without immunosuppressive therapy; 15 patients required glucocorticosteroids for hepatotoxicity-related AEs. Overall, a low incidence of irAEs was observed. CRS was the most common irAE reported, occurring in 17 patients. In an additional 14 patients, other irAEs observed were immune-mediated hepatitis/acute hepatitis, rash, stomatitis, dermatitis, arthritis, uveitis, pneumonitis, myalgia, and arthralgia and occurred in ≤4 patients each.

TEAEs leading to discontinuation of INBRX-105 were reported in 20 patients (24.7%) who received monotherapy and 4 patients (5.1%) who received the combination. The most common TEAEs leading to discontinuation (≥3 of all enrolled patients) were increased AST [*n* = 6 (grade ≥3, *n* = 4); INBRX-105–related, *n* = 5 (grade ≥3, *n* = 4)], increased ALT [*n* = 4 (grade ≥3, *n* = 3); all INBRX-105–related], and immune-mediated hepatitis [*n* = 3 (grade ≥3, *n* = 2); all INBRX-105–related].

Serious AEs (SAE) occurred in 59.3% and 46.8% of patients who received INBRX-105 monotherapy and combination therapy, respectively. The most common SAEs overall were sepsis (monotherapy, 3.7%; combination, 6.3%) and CRS (1.2%; 7.6%). INBRX-105–related SAEs were reported in 18.5% of patients who received monotherapy and 13.9% of patients who received the combination. The most common treatment-related SAE was immune-mediated hepatitis with monotherapy (*n* = 4; 4.9%) and CRS with the combination (*n* = 6; 7.6%). Nine patients had TEAEs resulting in death (monotherapy, *n* = 3; combination, *n* = 6); no deaths were related to hepatic events (Supplementary Table S5). One patient had a treatment-related TEAE that resulted in death (CRS, occurring in the combination subgroup; Supplementary Table S3).

### PK and immunogenicity

**Figure 1.**
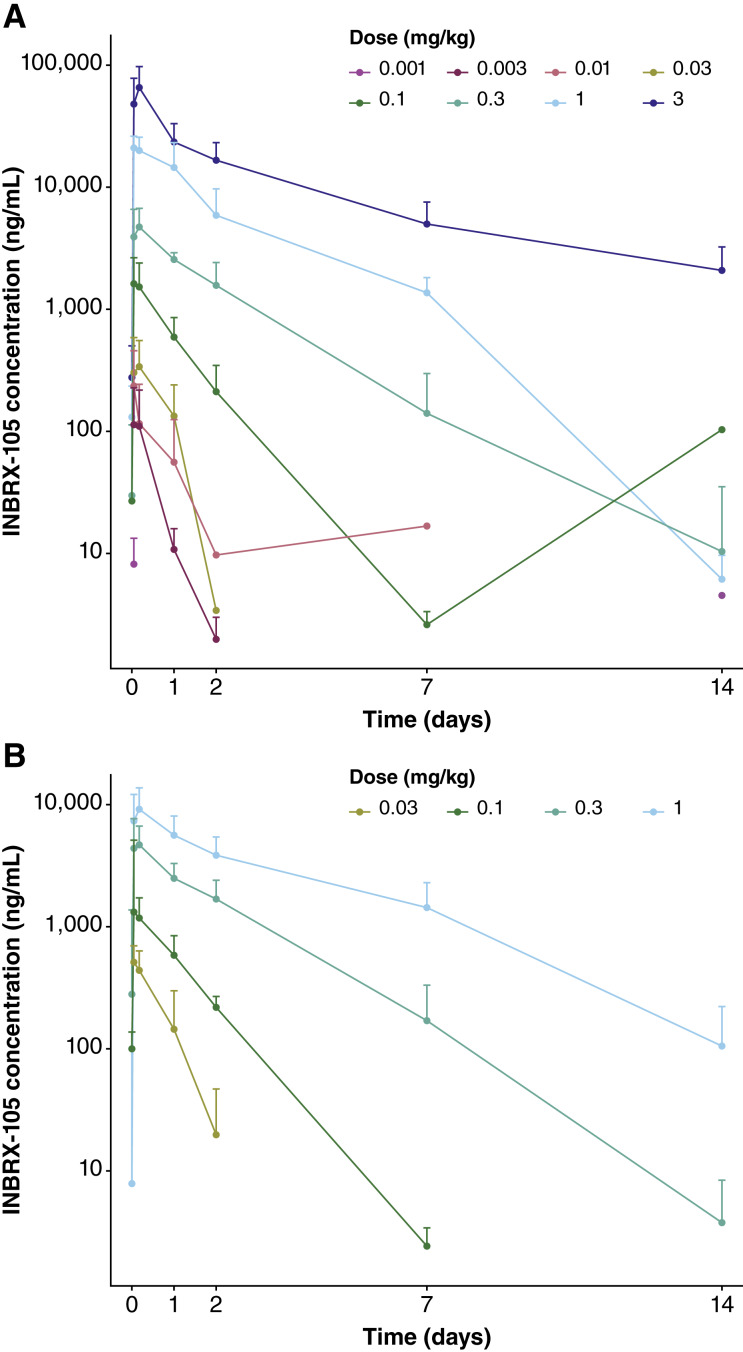
PK profile of INBRX-105 monotherapy (**A**) and INBRX-105 in combination with pembrolizumab (**B**). Mean serum concentrations of INBRX-105 over time during cycle 1 are shown. Error bars indicate standard deviation.

PK data from 153 patients (monotherapy, *n* = 75; combination, *n* = 78) were analyzed. Dose-related increases in mean serum INBRX-105 C_max_ values were observed and were approximately dose-proportional at the higher doses administered (Fig. 1A and B; Supplementary Table S6). The mean half-life of INBRX-105 ranged from 0.3 to 3.75 days across the dose levels examined.

Of the 132 patients evaluable for ADAs both before and during dosing, 51 (38.6%) were positive for ADAs to INBRX-105. No apparent difference in disease progression profiles were observed in patients who developed ADAs and those who did not. Overall, the incidence or magnitude of ADAs to INBRX-105 did not significantly differ across dose levels or in combination with pembrolizumab. ADAs to INBRX-105 were most commonly first detectable after the first two treatment cycles but were not associated with SAEs. INBRX-105 exposure was generally lower in patients with ADAs than in patients without, although the degree of reduced exposure varied. However, ADAs, and the resultant loss of exposure, were not associated with infusion-related reactions or SAEs, including those that were liver-related. Furthermore, exploratory assessments showed that ADA positivity was not associated with immediate disease progression. In a subset of 25 patients positive for INBRX-105 ADAs, 24 had neutralizing antibodies at ≥1 time point. Of 152 patients evaluable for ADAs prior to dosing, only one patient (0.7%) was positive for preexisting ADAs.

### Efficacy

**Figure 2.**
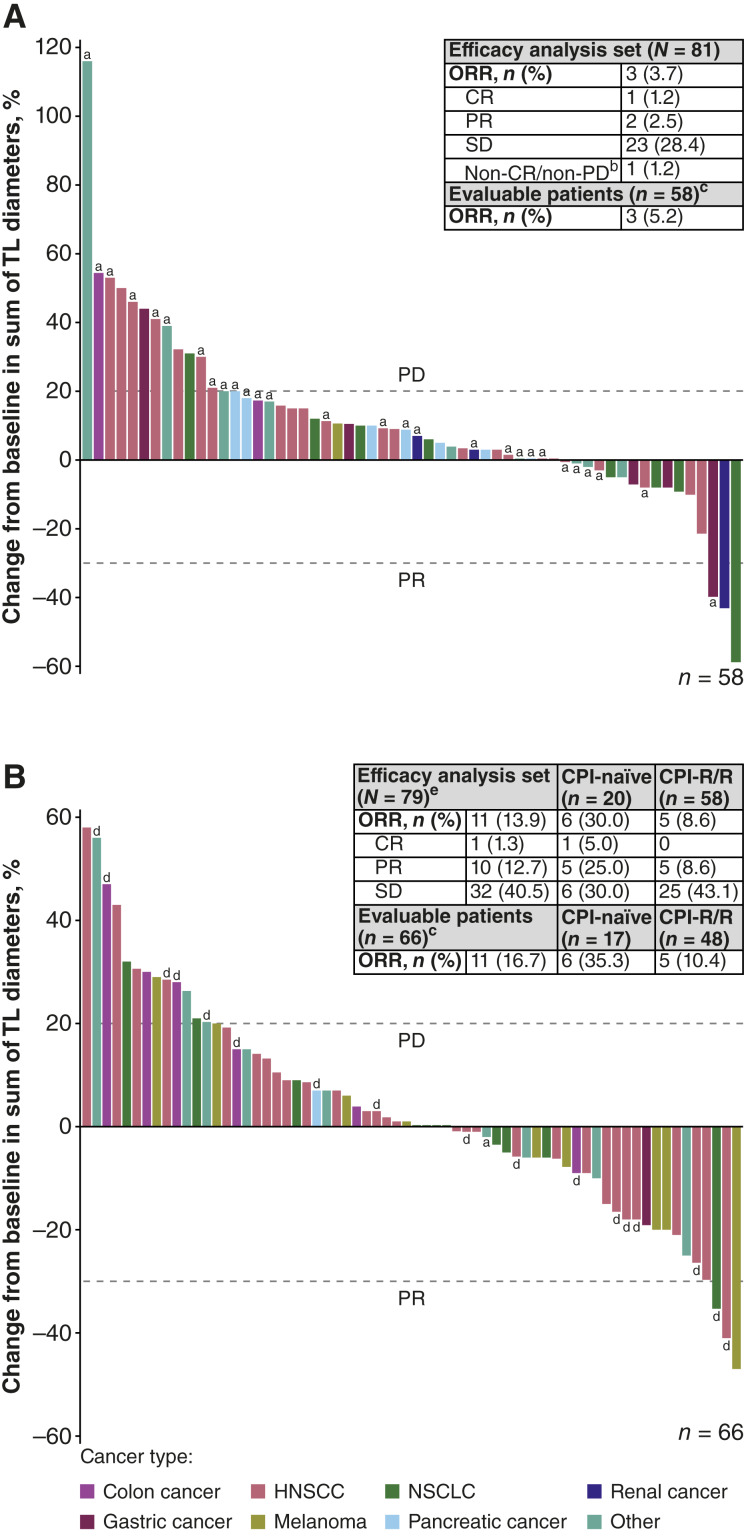
Best change from baseline in size of target lesion in patients who received INBRX-105 alone (**A**) or in combination with pembrolizumab (**B**). Enrolled patients had CPI-R/R disease unless otherwise indicated (see footnotes a and d). The ORR was the proportion of patients with a BOR of CR or PR by RECIST 1.1. The waterfall plots present the best postbaseline target lesion response in evaluable patients (i.e., patients with ≥1 postbaseline assessment who also had measurable target disease at baseline). ^a^Patient with prior CPI receipt unknown. ^b^Applicable only to patients with nonmeasurable disease (i.e., nontarget disease) at baseline. ^c^Evaluable patients were all patients with ≥1 postbaseline assessment who also had measurable target disease at baseline. ^d^Patient with CPI-naïve disease. ^e^A patient with an unknown prior CPI status and a BOR of SD was also included in the total efficacy (*N* = 79) and evaluable patients (*n* = 66) analyses. BOR, best overall response; PD, progressive disease; SD, stable disease; TL, target lesion.

The ORR in all patients (*N* = 160) was 8.8%, and the DCR was 43.1%. The ORR in patients who received INBRX-105 monotherapy (*n* = 81) was 3.7% [CR, *n* = 1 (CPI-R/R; monotherapy dose expansion, INBRX-105 dose, 0.3 mg/kg); PR, *n* = 2 (CPI-R/R, *n* = 1; monotherapy dose expansion, INBRX-105 dose: 0.3 mg/kg, *n* = 1; 1 mg/kg, *n* = 1); Fig. 2A; Supplementary Table S7], and the DCR was 32.1%. In the 58 patients with evaluable disease (i.e., ≥1 postbaseline assessment and measurable target disease at baseline) who received monotherapy, the ORR was 5.2% and the DCR was 44.8%.

In all patients who received INBRX-105 in combination with pembrolizumab (*n* = 79), the ORR was 13.9% [CR, *n* = 1 (combination dose expansion, INBRX-105: 0.3 mg/kg); PR, *n* = 10 (combination dose escalation, INBRX-105 dose: 0.03 mg/kg, *n* = 1; 0.3 mg/kg, *n* = 2; 1 mg/kg, *n* = 1; combination dose expansion, INBRX-105 dose: 0.3 mg/kg, *n* = 6)] and 16.7% in evaluable patients (*n* = 66; Fig. 2B; Supplementary Table S7). ORRs were also evaluated by CPI status in those who received combination treatment; 20 patients had CPI-naïve disease (evaluable, *n* = 17), and 58 patients CPI-R/R disease (evaluable, *n* = 48). The ORR among patients with CPI-naïve disease was 30% (evaluable, 35.3%), with five PRs (CPS/TPS <20, *n* = 3; CPS/TPS missing, *n* = 2) and 1 CR (CPS/TPS ≥20). The ORR among patients with CPI-R/R disease was 8.6% (evaluable, 10.4%), all PRs (*n* = 5; CPS/TPS <20, *n* = 3; CPS/TPS ≥20, *n* = 1; CPS/TPS missing, *n* = 1). In patients with CPI-naïve versus CPI-R/R disease, the DCR was 60% (evaluable, 70.6%) versus 51.7% (evaluable, 62.5%).

The duration of response for 10 of the 14 responders (71.4%) was <6 months (<2 months, *n* = 6). Among the other four patients, all of whom received the combination, the duration of response ranged from 7.2 to 15.4 months.

## Discussion

In this phase 1 study of INBRX-105, most treatment-related TEAEs were grade 1 or 2. Grade ≥3 treatment-related TEAEs tended to be liver-related (e.g., increased AST and ALT) and reversed rapidly. INBRX-105–related TEAEs that led to treatment discontinuation were also most frequently liver-related. One death due to a treatment-related TEAE (CRS) occurred in the combination subgroup.

The PK analyses showed that INBRX-105 exposure was similar, whether the drug was administered as monotherapy or in combination with pembrolizumab. The terminal elimination half-life at the higher doses was approximately 3.75 days and was similar to that previously reported for other PD-L1×4-1BB bispecific antibodies (e.g., MCLA-145, 2.9 days at 75 mg dose; acasunlimab, 2.3–10.3 days across doses evaluated; refs. 16, 20). Preliminary analyses suggested that anti–INBRX-105 ADAs reduced INBRX-105 exposure.

Although INBRX-105 was designed to localize 4-1BB agonism to regions of high PD-L1 expression (e.g., the tumor microenvironment) with the goal of reducing liver-related AEs, hepatotoxicity was still observed, particularly at higher doses. This was consistent with what had been observed with other 4-1BB agonistic antibodies in preclinical studies in mice (21–23) and the safety profile reported for 4-1BB agonists in clinical development, such as urelumab. In clinical trials, higher doses of urelumab were associated with an increased incidence of hepatotoxicity (11), leading to a reduced dose being examined in combination with other immunotherapeutics. Similar findings were observed in a phase 2 trial of the PD-L1×4-1BB bispecific antibody acasunlimab alone or in combination with pembrolizumab in patients with previously treated metastatic NSCLC. In this study, treatment-related hepatic AEs were reported in 13.6% of patients in the monotherapy arm (grade ≥3, 9.1%) and 28.6% of patients in the combination once every 3 weeks dosing arm (grade ≥3, 16.7%; ref. 12). Less-frequent dosing (i.e., acasunlimab once every 6 weeks) was associated with a lower incidence of treatment-related hepatic AEs (any grade, 18.4%; grade ≥3, 12.2%). Most of the AEs leading to treatment discontinuation were asymptomatic hepatic events that resolved with corticosteroids and/or dose delays. Similarly, early findings from a phase 1 study of the PD-L1×4-1BB bispecific MCLA-145 reported ALT/AST increase as one of the most common AEs, with an incidence of 25% (grade 3/4, 11%) in patients who received monotherapy and 21% (grade 3/4, 11%) in patients who received MCLA-145 in combination with pembrolizumab (16). The molecular basis for such observations may involve activation of 4-1BB on liver myeloid cells (e.g., Kupffer cells), which triggers the secretion of proinflammatory cytokines and, consequently, hepatotoxicity (10).

Although TEAEs were generally low grade, the clinical activity of INBRX-105 was limited (ORR in all patients, 8.8%), and most patients did not have durable responses (duration of response >6 months, 28.6%). When INBRX-105 was combined with pembrolizumab, greater efficacy was observed, but the ORR remained modest (≤15%). This was similar to findings from a recent phase 2 study, in which acasunlimab showed greater efficacy when combined with pembrolizumab in the CPI-R/R setting (12). Although cross-trial comparisons must be made with caution, the unconfirmed ORR in the acasunlimab once every 6 weeks combination arm (29.6%) was considerably higher (12) than that observed with INBRX-105 in a similar patient population (CPI-R/R, 10.4% in evaluable patients). The ORR in evaluable patients with CPI-naïve disease who received the INBRX-105 combination was 35.3%. The extent to which first-time exposure to pembrolizumab may have contributed to this increased response rate in patients with CPI-naïve disease is difficult to determine.

The low response rate and hepatic safety signals observed with INBRX-105 did not demonstrate sufficient clinical promise to warrant further exploration. As with other clinical-stage 4-1BB agonists, based on the benefit–risk ratio observed in this study, clinical development of INBRX-105 has been discontinued. Additional treatment options for patients with tumors refractory to CPIs remain an important unmet need.

## Supplementary Material

Figure S1 Figure S1 shows that in vitro INBRX-105 blocks PD-1 binding of PD-L1, induces signaling downstream of 4-1BB in a PD-L1–dependent manner, and has greater binding affinity for PD-L1 vs 4-1BB

Figure S2 Figure S2 shows that in vitro INBRX-105 resulted in greater T-cell modulation than a constitutive 4-1BB agonist that was provided alone or in combination with a PD-L1 antagonist

Figure S3 Figure S3 shows that INBRX-105-a, a murine INBRX-105 surrogate, demonstrated potent antitumor activity, including T-cell tumor infiltration and induction of T-cell memory, in PD-L1+ mouse tumor models

Figure S4 Figure S4 shows that addition of an orthogonal anti–PD-1 antibody to INBRX-105-a resulted in enhanced antitumor activity in mouse tumor models and improved blockade of PD-1/PD-L1 interactions compared with INBRX-105-a alone

Figure S5 Figure S5 is a schematic of the study design

Table S1 Table S1 summarizes the representativeness of study participants

Table S2 Table S2 shows patient disposition

Table S3 Table S3 summarizes the treatment-emergent adverse events

Table S4 Table S4 summarizes the INBRX-105–related hepatic treatment-emergent adverse events

Table S5 Table S5 summarizes the treatment-emergent adverse events that resulted in death

Table S6 Table S6 shows the pharmacokinetic parameter estimates for INBRX-105 in cycle 1

Table S7 Table S7 summarizes the characteristics of patients who were responders to study treatment

## Data Availability

The data generated and/or analyzed in this study are not publicly available but are available upon reasonable request. Sharing of data is subject to protection of patient privacy and respect for the patient’s informed consent. For more information on the process or to submit a request, contact clinicaltrials@inhibrx.com.
