# Supplementary material for: Phase 1 Study of INBRX-105, a TNFRSF9 (4-1BB) and PD-L1 Bispecific Antibody, in Patients with Select Solid Tumors
Source: Cancer Res Commun. 2026 Feb 23;6(2):374–82. doi: 10.1158/2767-9764.CRC-25-0577 (PMC13143200; doi:10.1158/2767-9764.CRC-25-0577)
Supplement: Figure S1 — shows that in vitro INBRX-105 blocks PD-1 binding of PD-L1, induces signaling downstream of 4-1BB in a PD-L1–dependent manner, and has greater binding affinity for PD-L1 vs 4-1BB [file crc-25-0577_figure_s1_suppsf1.pdf]

Supplementary Figure S1. INBRX-105 blocks PD-L1 and demonstrates PD-L1–dependent 4-1BB agonism.

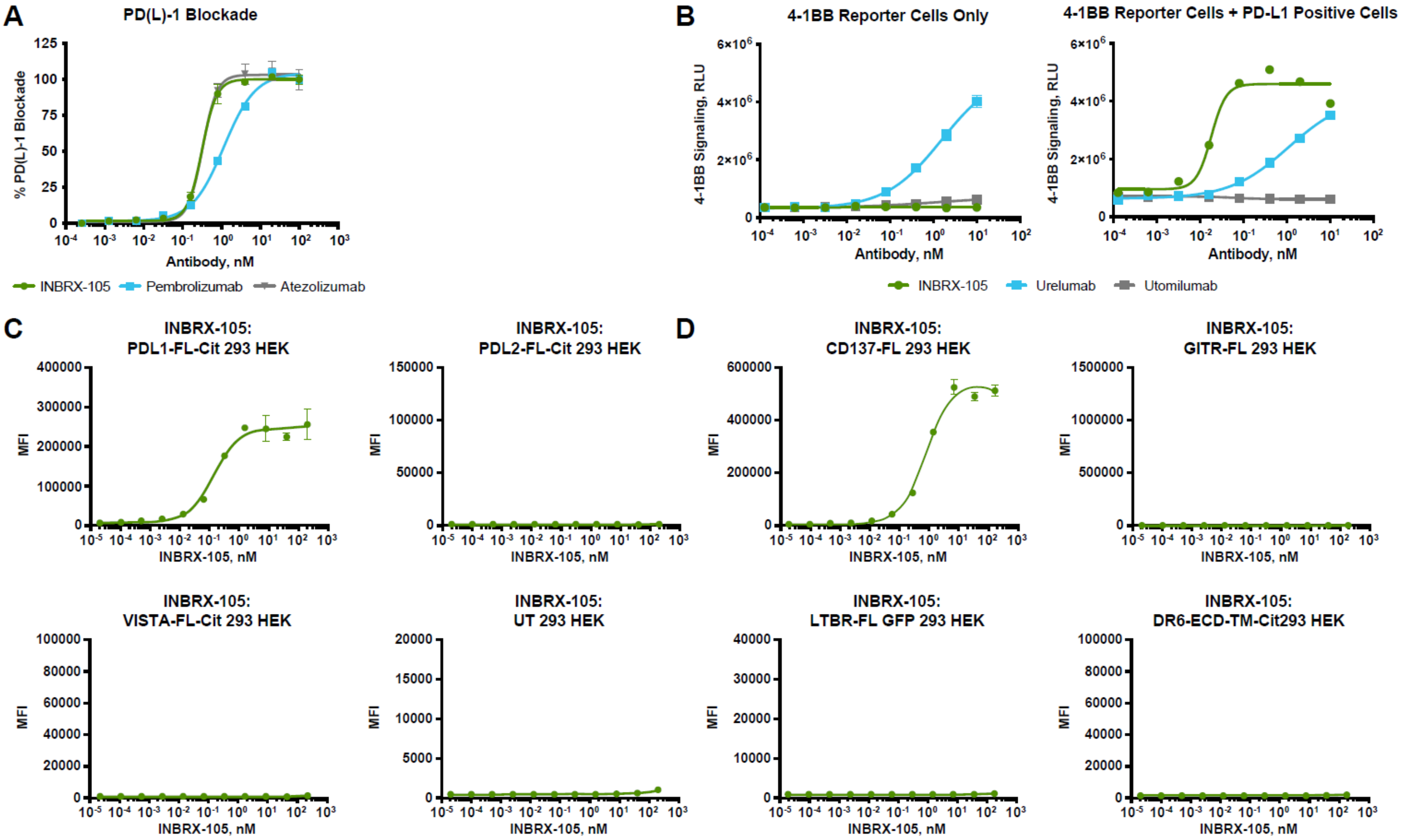

(A) Evaluation of PD-1/PD-L1 blockade in a Promega PD-1/PD-L1 blockade reporter assay. PD-1/PD-L1 blockade reporter cells (Promega) were incubated with INBRX-105, atezolizumab (anti–PD-L1), or pembrolizumab (anti–PD-1), and percent blockade was determined relative to the maximum luciferase signal. **(B) INBRX-105 activity is dependent on PD-L1 expression.** The agonist activity of INBRX-105 was determined in a luciferase-based 4-1BB activation assay in the presence or absence of PD-L1$^+$ K562 cells. The agonist activity of INBRX-105 was found to be PD-L1–dependent and absent without PD-L1$^+$ K562 cells. In contrast, the constitutive 4-1BB agonist analogue of urelumab (synthesized based on publicly disclosed sequences) showed PD-L1–independent activity, while the Fc-crosslinking–dependent analogue of utomilumab (synthesized based on publicly disclosed sequences) showed no activity. **Binding affinity of INBRX-105 to PD-L1, 4-1BB, and related TNFRSF and B7 family members. (C) Titration FACS of INBRX-105 on PD-L1 and related B7 family member–expressing HEK293 cells, including an untransfected cell comparator.** Detection in the 647 nm channel as an indirect measurement of INBRX-105 binding was only seen on PD-L1–expressing cells, with an apparent affinity of 0.15 nM (blue curve, top left). All other populations were negative, including untransfected cells. Symbols are the mean of experimental duplicates, and the error bars reflect the range detected. **(D) Titration FACS of INBRX-105 on 4-1BB and related TNFRSF protein family member–expressing HEK293 cells.** Detection in the 647 nm channel as an indirect measurement of INBRX-105 binding was only seen on 4-1BB (CD137) expressing cells, with an apparent affinity of 0.92 nM (blue curve, top left). All other populations were negative. Symbols are the mean of experimental duplicates, and the error bars reflect the range detected.

Panels A and B originally from Kinkead H, et al. Presented at the 2021 SITC Annual Meeting. Abstract 12. Reprinted with permission from the author.

Abbreviations: Cit, citrine; ECD, extracellular domain; FACS, fluorescence-activated cell sorting; FL, full length; GFP, green fluorescent protein; MFI, mean fluorescence intensity; RLU, relative luminescence units; TM, transmembrane domain; TNFRSF, tumor necrosis factor receptor superfamily; UT, untransfected.
