# Supplementary material for: Phase 1 Study of INBRX-105, a TNFRSF9 (4-1BB) and PD-L1 Bispecific Antibody, in Patients with Select Solid Tumors
Source: Cancer Res Commun. 2026 Feb 23;6(2):374–82. doi: 10.1158/2767-9764.CRC-25-0577 (PMC13143200; doi:10.1158/2767-9764.CRC-25-0577)
Supplement: Figure S2 — shows that in vitro INBRX-105 resulted in greater T-cell modulation than a constitutive 4-1BB agonist that was provided alone or in combination with a PD-L1 antagonist [file crc-25-0577_figure_s2_suppsf2.pdf]

Supplementary Figure S2. PD-L1–dependent 4-1BB agonism by INBRX-105 enhances T-cell activity over combination treatment.

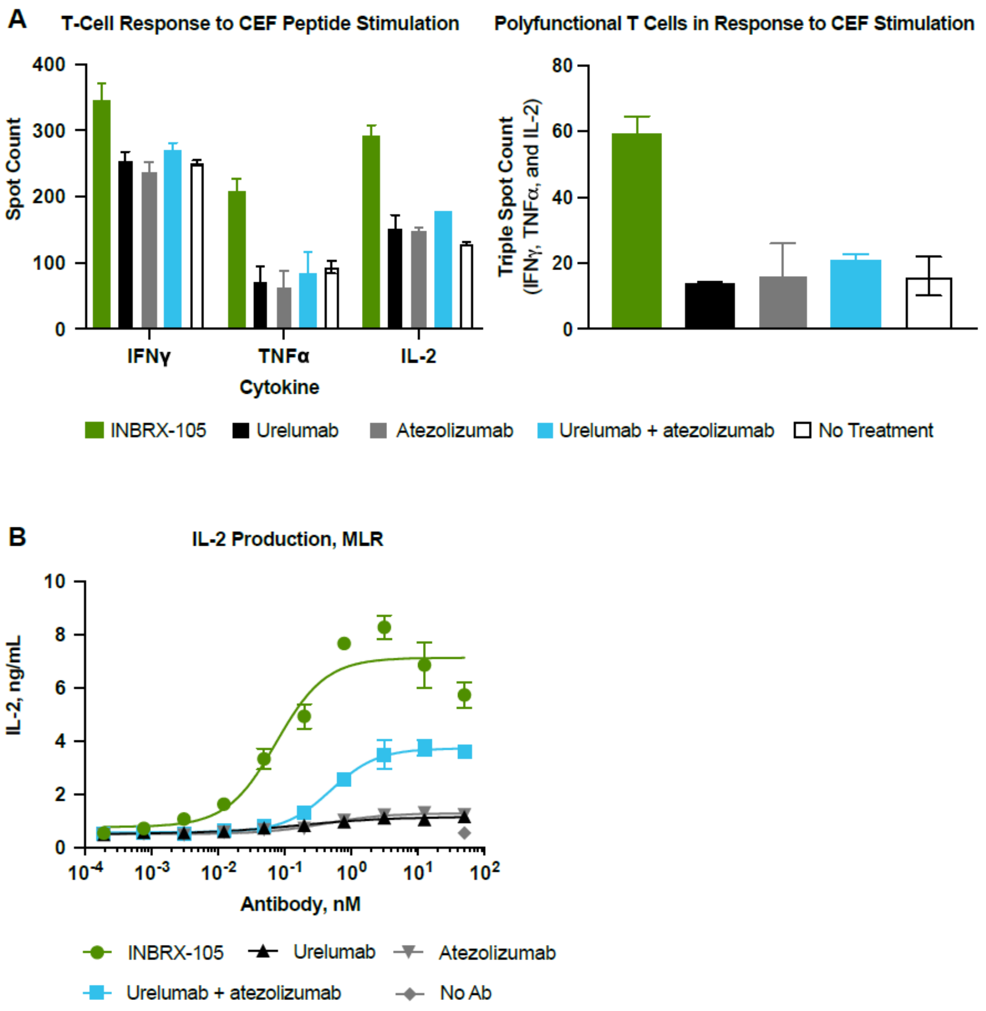

Cytokine production was enhanced in T cells when 4-1BB co-stimulation was provided via PD-L1 localization as compared with 4-1BB blockade (urelumab; synthesized based on publicly disclosed sequences) in the presence or absence of PD-L1 blockade (atezolizumab) provided by an independent antibody. **(A)** PBMCs were cultured with CEF (CMV, influenza, and EBV) peptides to elicit antigen-specific T-cell activation and cytokine production was assessed using IFNγ/TNFα/IL-2 three-color FluoroSpot assay. **(B)** Immature dendritic cells and T cells were co-cultured in an allogeneic mixed lymphocyte reaction. IL-2 concentrations in the culture supernatant were determined by ELISA.

Panels originally from Kinkead H, et al. Presented at the 2021 SITC Annual Meeting. Abstract 12. Reprinted with permission from the author.

Abbreviations: Ab, antibody; CMV, cytomegalovirus; EBV, Epstein–Barr virus; ELISA, enzyme-linked immunosorbent assay; MLR, mixed lymphocyte reaction; PBMC, peripheral blood mononuclear cell.
