# Supplementary material for: Phase 1 Study of INBRX-105, a TNFRSF9 (4-1BB) and PD-L1 Bispecific Antibody, in Patients with Select Solid Tumors
Source: Cancer Res Commun. 2026 Feb 23;6(2):374–82. doi: 10.1158/2767-9764.CRC-25-0577 (PMC13143200; doi:10.1158/2767-9764.CRC-25-0577)
Supplement: Figure S3 — shows that INBRX-105-a, a murine INBRX-105 surrogate, demonstrated potent antitumor activity, including T-cell tumor infiltration and induction of T-cell memory, in PD-L1+ mouse tumor models [file crc-25-0577_figure_s3_suppsf3.pdf]

Supplementary Figure S3. 4-1BB agonism induces T cell memory; efficacy requires both 4-1BB agonism and PD-L1 blockade.

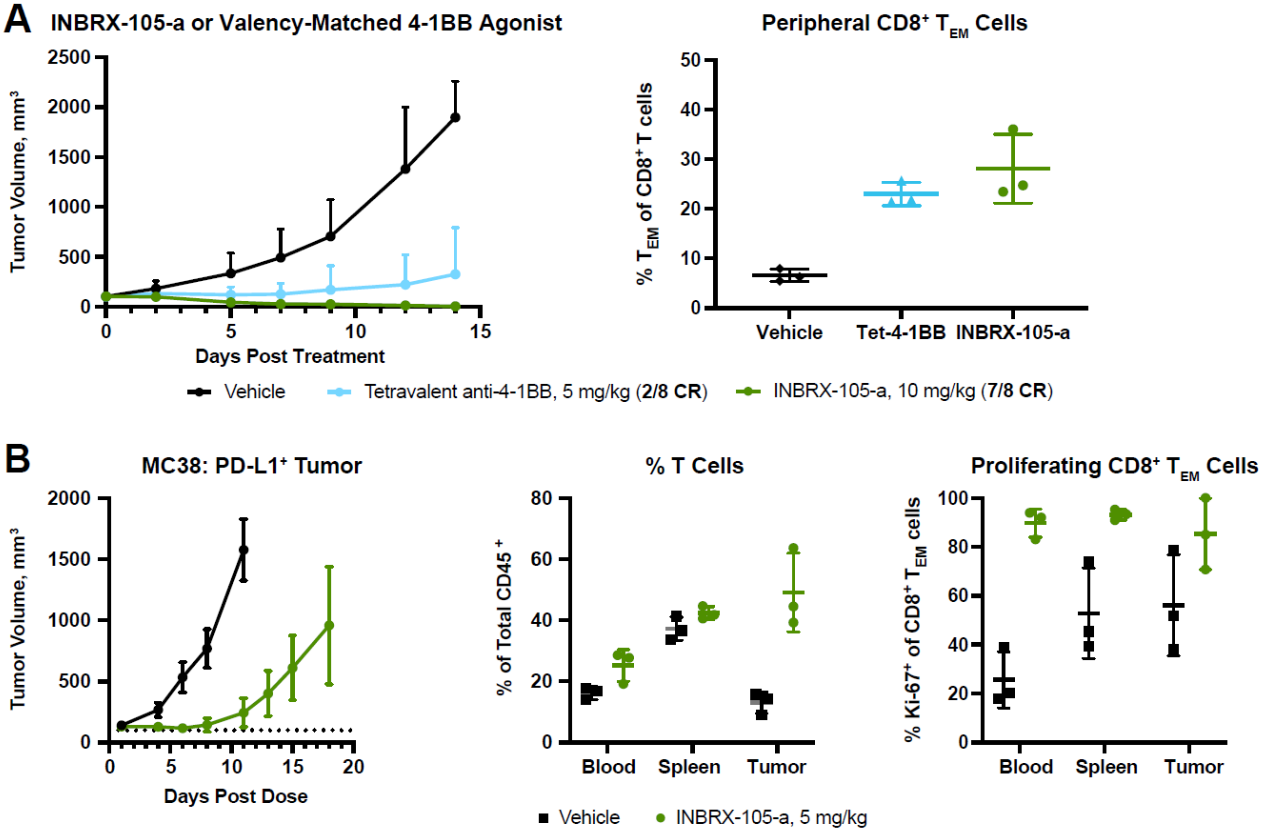

(A) A surrogate molecule (INBRX-105-a) matched for format, affinity, and potency to INBRX-105 is superior to a dose-matched tetravalent 4-1BB agonist lacking PD-L1 targeting. C57BL/6 mice bearing subcutaneous MC38 tumors were treated once tumors reached 100 ± 20 mm^3. Peripheral T cells were analyzed on day 7 post dose. **(B)** C57BL/6 mice bearing subcutaneous MC38 tumors were treated with a surrogate molecule (INBRX-105-a) once tumors reached 100 ± 20 mm^3. Peripheral and intratumoral T cell populations were analyzed on day 7 post dose. INBRX-105-a demonstrates antitumor efficacy coupled with increased T-cell infiltration into tumors as well as robust proliferation of $CD8^+$ T_{EM} cells (defined as $CD44^+CD62L^-$) within the $CD8^+$ T-cell compartment.

Figure originally from Kinkead H, et al. Presented at the 2021 SITC Annual Meeting. Abstract 12. Reprinted with permission from the author.

Abbreviations: CR, complete response; T_{EM}, T effector memory cell; tet-4-1BB, tetravalent 4-1BB agonist.
