# Supplementary material for: Phase 1 Study of INBRX-105, a TNFRSF9 (4-1BB) and PD-L1 Bispecific Antibody, in Patients with Select Solid Tumors
Source: Cancer Res Commun. 2026 Feb 23;6(2):374–82. doi: 10.1158/2767-9764.CRC-25-0577 (PMC13143200; doi:10.1158/2767-9764.CRC-25-0577)
Supplement: Figure S4 — shows that addition of an orthogonal anti–PD-1 antibody to INBRX-105-a resulted in enhanced antitumor activity in mouse tumor models and improved blockade of PD-1/PD-L1 interactions compared with INBRX-105-a alone [file crc-25-0577_figure_s4_suppsf4.pdf]

Supplementary Figure S4. PD-1 blockade increases the potency of INBRX-105 to achieve maximal efficacy.

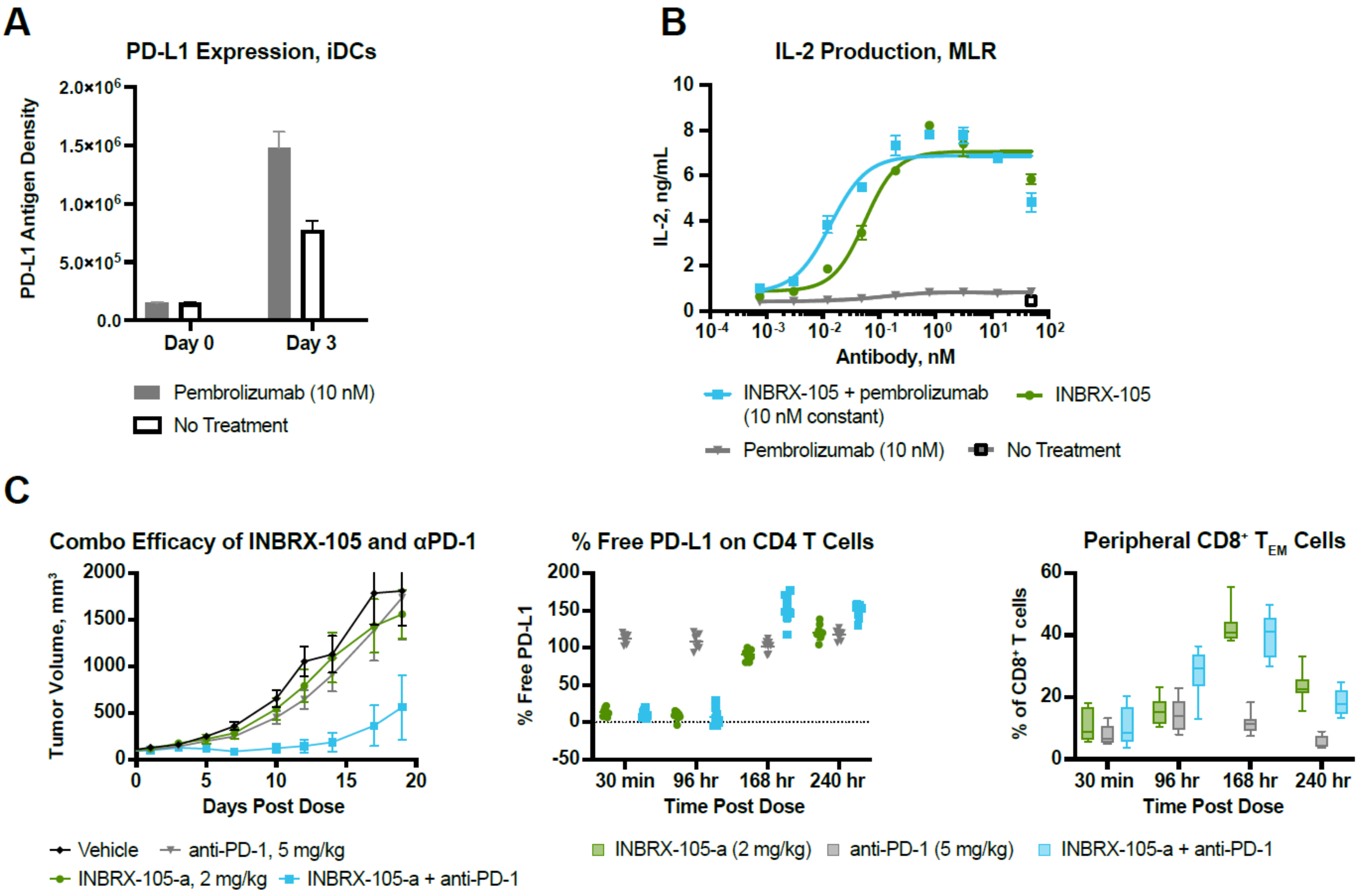

(A) PD-L1 antigen density of in vitro derived immature dendritic cells from an MLR was quantified by flow cytometry. **(B)** In vitro, the addition of orthogonal PD-1 blockade provided by pembrolizumab increases the potency of INBRX-105. In vitro derived immature dendritic cells and HLA mismatched T cells were co-cultured in an allogeneic mixed lymphocyte reaction to induce T-cell activation, which was measured by ELISA for IL-2 production. **(C)** C57BL/6 mice bearing subcutaneous MC38 tumors were dosed with 2 mg/kg INBRX-105-a, sufficient to induce maximal pharmacodynamic activity (driven by 4-1BB agonism and as determined by expansion of $CD8^+$ T_{EM} cells), and 5 mg/kg αPD-1 to induce optimal anti-tumor activity without changing occupancy or pharmacodynamic biomarkers.
Figure originally from Kinkead H, et al. Presented at the 2021 SITC Annual Meeting. Abstract 12. Reprinted with permission from the author.
Abbreviations: ELISA, enzyme-linked immunosorbent assay; iDC, immature dendritic cell; MLR, mixed lymphocyte reaction; T_{EM}, T effector memory cell.
