# Supplementary material for: Phase 1 Study of INBRX-105, a TNFRSF9 (4-1BB) and PD-L1 Bispecific Antibody, in Patients with Select Solid Tumors
Source: Cancer Res Commun. 2026 Feb 23;6(2):374–82. doi: 10.1158/2767-9764.CRC-25-0577 (PMC13143200; doi:10.1158/2767-9764.CRC-25-0577)
Supplement: Figure S5 — is a schematic of the study design [file crc-25-0577_figure_s5_suppsf5.pdf]

Supplementary Figure S5. Study design.

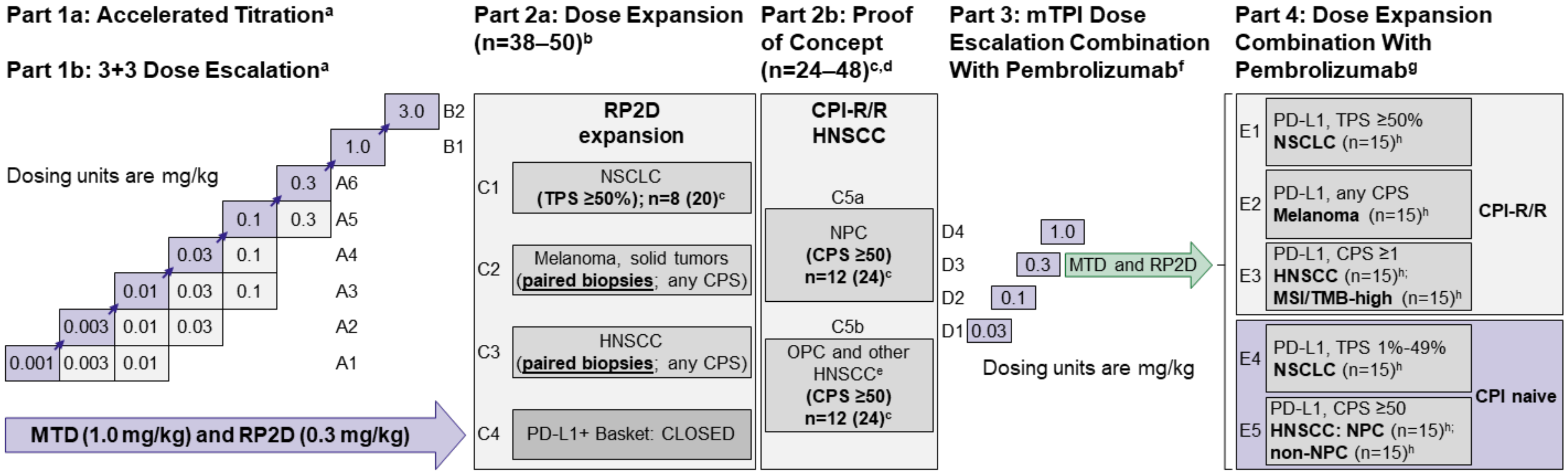

In parts 1 and 2, a cycle was defined as 28 days. In part 1, the dose-limiting toxicity (DLT) window was the first treatment cycle (28 days). For patients in part 1a who escalated dose levels, the DLT window was cycles 1 and 2. In part 2, a DLT window was not specified for part 2 in the protocol, but toxicities associated with the initial RP2D of 1 mg/kg were designated as DLTs. The dose of INBRX-105 was subsequently reduced to 0.3 mg/kg. In parts 3 and 4, INBRX-105 was administered as an IV infusion over 60 minutes Q3W; a cycle was defined as 21 days. The DLT window in part 3 was the first treatment cycle.

[a] Part 1 used a traditional 3+3 design. INBRX-105 was administered as an IV infusion over 60 minutes Q2W at the doses shown in squares. Intrapatient dose escalations are depicted in gray. In part 1a, up to 6 patients were included per cohort. In part 1b, 3 to 6 patients were included per cohort. Cohorts are labeled as A1-A6 and B1-B2. [b] In part 2a, the first 6 patients received INBRX-105 at a dose of 1.0 mg/kg Q2W, and the second 16 patients received a dose of 0.3 mg/kg Q3W. The remaining patients in cohorts C1, C2, and C3 received INBRX-105 at a dose of 0.3 mg/kg Q4W. [c] Per SRC recommendation, any cohort or subcohort could be expanded to a total of 24 patients (including the last 4 to 6 patients with paired biopsies). A PD-L1 CPS >20 was allowed in the expansion. [d] In part 2b, patients received INBRX-105 at the MTD and RP2D of 0.3 mg/kg Q4W. [e] OPC HNSCC or other locations/types (larynx, [hypo]pharynx, or sinus). [f] In part 3, PD-L1 expression by IHC was not required. Each cohort (D1-D4) had

≥3 patients. [g] In part 4, INBRX-105 was administered at the MTD/RP2D. Cohorts are labeled as E1-E5. [h] Per SRC recommendation, any cohort or subcohort could be expanded to a total of 25 patients.

Abbreviations: CPI, checkpoint inhibitor; CPS, combined positive score; DLT, dose-limiting toxicity; HNSCC, head and neck squamous cell carcinoma; IHC, immunohistochemistry; IV, intravenous; MSI, microsatellite instability; MTD, maximum tolerated dose; mTPI, modified toxicity probability interval; NPC, nasopharyngeal carcinoma; NSCLC, non-small cell lung cancer; OPC, oropharyngeal carcinoma; PD-L1, programmed cell death 1 ligand 1; Q2W, every 2 weeks; Q3W, every 3 weeks; Q4W, every 4 weeks; RP2D, recommended phase 2 dose; R/R, relapsed/refractory; SRC, scientific review committee; TMB, tumor mutation burden; TPS, tumor proportion score
