# Supplementary material for: Phase 1 Study of INBRX-105, a TNFRSF9 (4-1BB) and PD-L1 Bispecific Antibody, in Patients with Select Solid Tumors
Source: Cancer Res Commun. 2026 Feb 23;6(2):374–82. doi: 10.1158/2767-9764.CRC-25-0577 (PMC13143200; doi:10.1158/2767-9764.CRC-25-0577)
Supplement: Table S1 — summarizes the representativeness of study participants [file crc-25-0577_table_s1_suppst1.docx]

**Supplementary Table S1. Representativeness of study participants**

| Cancer type(s)/subtype(s)/stage(s)/condition | Locally advanced or metastatic unresectable solid tumors. The most common among patients in this study were non-small cell lung cancer (NSCLC; 15.6%) and head and neck squamous cell carcinoma (HNSCC; 38.1%). |
| --- | --- |
| **Considerations related to:** | |
| Sex | Each year, in the US, there are an estimated 483.5 new cases of cancer (any site) per 100,000 men and 421.3 new cases per 100,000 women (1). The incidence of NSCLC is slightly higher in men than women (men, 53.6%; women, 46.4%) (2). HNSCC is more common in men (men, 73.2%; women, 26.8%) (3). |
| Age | In the US, the median age at the time of diagnosis for all cancer types is 67 years (4). For lung cancer overall, the median age at diagnosis is 71 (5). For HNSCC, the median age at diagnosis is 64 (6). |
| Race/ethnicity | The highest incidences of cancer (any site) in the US are in individuals who identify as non-Hispanic White (477.9 cases per 100,000 people) or non-Hispanic Black (458.2 cases per 100,000 people) (7). In the US, most patients with NSCLC (75.2%) identify as White, 12.1% as Black, 6.3% as Asian/Pacific Islander, 5.8% as Hispanic, and 0.5% as American Indian/Alaska Native (2). Most patients with HNSCC (73.4%) identify as White, 11.5% as Black, 6.7% as Asian/Pacific Islander, 7.3% as Hispanic, and 0.7% as American Indian/Alaska Native (3). |
| Geography | In the US, the annual estimated incidence of lung and bronchus cancer is 47.8 new cases per 100,000 people; the annual estimated incidence of deaths is 31.5 per 100,000 people (5).  In 2020, it was estimated that there were 10.4 new cases of HNSCC and 2.1 deaths per 100,000 people in the US (6). |
| **Other considerations** | Up to 64% of patients with NSCLC have secondary resistance to checkpoint inhibitors (CPIs) (8). Only 20% to 30% of patients with HNSCC exhibit long-term benefit from treatment with immunotherapy (9). |
| **Overall representativeness of this study** | |
| This phase 1 study enrolled 160 patients aged ≥18 years across 17 sites in the US. Eligible patients had advanced or metastatic unresectable solid tumors whose disease had progressed despite standard therapy and for whom no further standard therapy exists or who refuse available standard treatment options. Multiple subcohorts specifically enrolled patients with NSCLC (relapsed/refractory to CPI [CPI-R/R] or CPI naive) or CPI-R/R HNSCC. Thus, HNSCC and NSCLC were the most common tumor types in this study (38.1% and 15.6% of all patients on study, respectively). Other solid tumor types included gastric, colon, pancreatic, and renal cancers, as well as melanoma and others (see **Table 1** in the main text).  The median age of patients on study (65 years for patients receiving monotherapy; 64 years for those receiving the combination) was similar to that reported for cancer overall (67 years). Consistent with the incidence of cancer overall in the US, most patients in this study were White (76.2%) and male (55.6%). | |

**Supplementary References**

1. Surveillance, Epidemiology, and End Results Program. Cancer Stat Facts: cancer of any site. [cited 2025 Aug 21]. Available from <https://seer.cancer.gov/statfacts/html/all.html>.

2. Primm KM, Zhao H, Hernandez DC, Chang S. Racial and ethnic trends and disparities in NSCLC. *JTO Clin Res Rep* 2022;**3**(8):100374.

3. Fakhry C, Krapcho M, Eisele DW, D'Souza G. Head and neck squamous cell cancers in the United States are rare and the risk now is higher among white individuals compared with black individuals. *Cancer* 2018;**124**(10):2125–33.

4. National Cancer Institute. Cancer causes and prevention: age and cancer risk. [cited 2025 Aug 21]. Available from <https://www.cancer.gov/about-cancer/causes-prevention/risk/age>.

5. Surveillance, Epidemiology, and End Results Program. Cancer Stat Facts: lung and bronchus cancer. [cited 2025 Aug 21]. Available from <https://seer.cancer.gov/statfacts/html/lungb.html>.

6. Barsouk A, Aluru JS, Rawla P, Saginala K, Barsouk A. Epidemiology, risk factors, and prevention of head and neck squamous cell carcinoma. *Med Sci (Basel)* 2023;**11**(2):42.

7. Surveillance, Epidemiology, and End Results Program. All cancer sites combined: SEER 5-year age-adjusted incidence rates, 2018-2022. [cited 2025 Aug 21]. Available from <https://seer.cancer.gov/statistics-network/explorer/application.html?site=1&data_type=1&graph_type=10&compareBy=race&chk_race_1=1&chk_race_6=6&chk_race_5=5&chk_race_4=4&chk_race_9=9&chk_race_8=8&chk_race_3=3&chk_race_2=2&series=9&sex=1&age_range=1&hdn_stage=101&advopt_precision=1&advopt_show_ci=on&hdn_view=0#resultsRegion0>.

8. Zhou S, Yang H. Immunotherapy resistance in non-small-cell lung cancer: from mechanism to clinical strategies. *Front Immunol* 2023;**14**:1129465.

9. Botticelli A, Cirillo A, Strigari L, Valentini F, Cerbelli B, Scagnoli S*, et al.* Anti-PD-1 and anti-PD-L1 in head and neck cancer: a network meta-analysis. *Front Immunol* 2021;**12**:705096.
