# Supplementary material for: Phase 1 Study of INBRX-105, a TNFRSF9 (4-1BB) and PD-L1 Bispecific Antibody, in Patients with Select Solid Tumors
Source: Cancer Res Commun. 2026 Feb 23;6(2):374–82. doi: 10.1158/2767-9764.CRC-25-0577 (PMC13143200; doi:10.1158/2767-9764.CRC-25-0577)
Supplement: Table S2 — shows patient disposition [file crc-25-0577_table_s2_suppst2.docx]

**Supplementary Table S2. Summary of patient disposition**

| **Patient disposition** | **INBRX-105**  **(n=81), n (%)** | **INBRX-105 + pembrolizumab**  **(n=79), n (%)** | **Total**  **(N=160),  n (%)** |
| --- | --- | --- | --- |
| **Safety analysis set^a^** | 81 (100) | 79 (100) | 160 (100) |
| **Efficacy analysis set^b^** | 81 (100) | 79 (100) | 160 (100) |
| **Completed treatment^c^** | 42 (51.9) | 55 (69.6) | 97 (60.6) |
| Documented disease progression | 38 (46.9) | 50 (63.3) | 88 (55.0) |
| Died on treatment | 4 (4.9) | 5 (6.3) | 9 (5.6) |
| **Discontinued treatment** | 39 (48.1) | 24 (30.4) | 63 (39.4) |
| Withdrawal request by patient | 6 (7.4) | 6 (7.6) | 12 (7.5) |
| AE | 16 (19.8) | 3 (3.8) | 19 (11.9) |
| Investigator discretion | 5 (6.2) | 6 (7.6) | 11 (6.9) |
| Other | 12 (14.8) | 9 (11.4) | 21 (13.1) |
| **Completed study^d^** | 17 (21.0) | 11 (13.9) | 28 (17.5)^e^ |
| **Discontinued study** | 58 (71.6) | 65 (82.3) | 123 (76.9)^e^ |
| AE | 0 | 1 (1.3) | 1 (0.6) |
| Death | 42 (51.9) | 39 (49.4) | 81 (50.6) |
| Loss to follow-up | 2 (2.5) | 2 (2.5) | 4 (2.5) |
| Physician decision | 1 (1.2) | 4 (5.1) | 5 (3.1) |
| Progressive disease | 0 | 2 (2.5) | 2 (1.3) |
| Sponsor request | 0 | 5 (6.3) | 5 (3.1) |
| Withdrawal by patient | 10 (12.3) | 9 (11.4) | 19 (11.9) |
| Other | 3 (3.7) | 3 (3.8) | 6 (3.8) |
| ^a^ The safety analysis set included all patients who received ≥1 dose of INBRX-105. ^b^ The efficacy analysis set included all patients who met entry criteria for enrollment regardless of whether they received study treatment. ^c^ Patients who experienced documented progressive disease and/or died are considered to have completed the study as the protocol-defined endpoint was observed in these patients. ^d^ Patients who completed all protocol-required follow-up activities and assessments or died. ^e^ The reason for study discontinuation was not recorded for a total of 9 patients. Therefore, these 9 patients are not characterized as having completed or discontinued the study (ie, they are omitted from these rows), and the percentages of patients completing the study or discontinuing from the study do not total 100%.  Abbreviation: AE, adverse event. | | | |
