# Supplementary material for: Phase 1 Study of INBRX-105, a TNFRSF9 (4-1BB) and PD-L1 Bispecific Antibody, in Patients with Select Solid Tumors
Source: Cancer Res Commun. 2026 Feb 23;6(2):374–82. doi: 10.1158/2767-9764.CRC-25-0577 (PMC13143200; doi:10.1158/2767-9764.CRC-25-0577)
Supplement: Table S3 — summarizes the treatment-emergent adverse events [file crc-25-0577_table_s3_suppst3.docx]

**Supplementary Table S3. Overall summary of TEAEs**

|  | **INBRX-105 (n=81), n (%)** | **INBRX-105 + pembrolizumab**  **(n=79), n (%)** |
| --- | --- | --- |
| **Patients with ≥1 TEAE** | **81 (100)** | **78 (98.7)** |
| SAE | 48 (59.3) | 37 (46.8) |
| Grade ≥3 | 61 (75.3) | 50 (63.3) |
| Leading to discontinuation of INBRX-105 | 20 (24.7) | 4 (5.1) |
| Leading to discontinuation of pembrolizumab | NA | 5 (6.3) |
| DLT | 8 (9.9) | 2 (2.5)^a^ |
| Resulting in death | 3 (3.7)^b^ | 6 (7.6)^c^ |
| **Patients with ≥1 INBRX-105–related TEAE** | **72 (88.9)** | **57 (72.2)** |
| SAE | 15 (18.5) | 11 (13.9) |
| Grade ≥3 | 30 (37.0) | 23 (29.1) |
| Leading to interruption of INBRX-105 | 9 (11.1) | 15 (19.0) |
| Leading to discontinuation of INBRX-105 | 14 (17.3) | 1 (1.3) |
| DLT | 8 (9.9) | 2 (2.5)^a^ |
| Resulting in death | 0 | 1 (1.3)^d^ |
| **Patients with pembrolizumab-related TEAEs** | **NA** | **47 (59.5)** |
| SAE | NA | 8 (10.1) |
| Grade ≥3 | NA | 18 (22.8) |
| Leading to interruption of pembrolizumab | NA | 4 (5.1) |
| Leading to discontinuation of pembrolizumab | NA | 1 (1.3) |
| DLT | NA | 1 (1.3)^a^ |
| Resulting in death | NA | 1 (1.3) |
| Related TEAEs were those with a relationship of possible, probable, or very likely/certainly related to INBRX-105/pembrolizumab as determined by the investigator. ^a^ These patients were in part 3 only; DLTs were not evaluated in part 4. ^b^ Urosepsis, multiple organ dysfunction syndrome, and tumor hemorrhage (each n=1). ^c^ Sepsis (n=2); urosepsis, coronavirus infection, multiple organ dysfunction syndrome, and respiratory failure (each n=1). ^d^ Cytokine release syndrome reported by the investigator as grade 3 with an outcome of death.  Abbreviations: DLT, dose-limiting toxicity; NA, not applicable; SAE, serious adverse event; TEAE, treatment-emergent adverse event. | | |
