# Supplementary material for: Phase 1 Study of INBRX-105, a TNFRSF9 (4-1BB) and PD-L1 Bispecific Antibody, in Patients with Select Solid Tumors
Source: Cancer Res Commun. 2026 Feb 23;6(2):374–82. doi: 10.1158/2767-9764.CRC-25-0577 (PMC13143200; doi:10.1158/2767-9764.CRC-25-0577)
Supplement: Table S4 — summarizes the INBRX-105–related hepatic treatment-emergent adverse events [file crc-25-0577_table_s4_suppst4.docx]

**Supplementary Table S4.** **INBRX-105–related hepatic TEAEs**

|  | **INBRX-105 (n=81), n (%)** | | | | | **INBRX-105 + pembrolizumab**  **(n=79), n (%)** | | | | | |
| --- | --- | --- | --- | --- | --- | --- | --- | --- | --- | --- | --- |
|  | **Grade** | | | | | **Grade** | | | | | |
|  | **1** | **2** | **3** | **4** | **5** | **1** | **2** | **3** | **4** | **5** |  |
| **Patients with ≥1 INBRX-105–related hepatic TEAE^a^** | 7 (8.6) | 7 (8.6) | 9 (11.1) | 2 (2.5) | 0 | 6 (7.6) | 4 (5.1) | 5 (6.3) | 1 (1.3) | 0 |  |
| AST increased | 8 (9.9) | 6 (7.4) | 6 (7.4) | 1 (1.2) | 0 | 5 (6.3) | 3 (3.8) | 4 (5.1) | 0 | 0 |  |
| ALT increased | 9 (11.1) | 1 (1.2) | 7 (8.6) | 0 | 0 | 4 (5.1) | 1 (1.3) | 5 (6.3) | 0 | 0 |  |
| Blood ALP increased | 4 (4.9) | 2 (2.5) | 3 (3.7) | 0 | 0 | 2 (2.5) | 1 (1.3) | 2 (2.5) | 0 | 0 |  |
| Blood bilirubin increased | 1 (1.2) | 2 (2.5) | 1 (1.2) | 0 | 0 | 0 | 0 | 0 | 0 | 0 |  |
| Transaminases increased | 0 | 1 (1.2) | 0 | 0 | 0 | 0 | 0 | 0 | 1 (1.3) | 0 |  |
| Blood ALP | 1 (1.2) | 0 | 0 | 0 | 0 | 0 | 0 | 0 | 0 | 0 |  |
| Immune-mediated hepatitis | 0 | 1 (1.2) | 3 (3.7) | 0 | 0 | 0 | 1 (1.3) | 0 | 0 | 0 |  |
| Hepatitis acute | 0 | 0 | 1 (1.2) | 0 | 0 | 0 | 0 | 1 (1.3) | 0 | 0 |  |
| Bile duct stenosis | 0 | 0 | 1 (1.2) | 0 | 0 | 0 | 0 | 0 | 0 | 0 |  |
| Gallbladder obstruction | 0 | 0 | 0 | 1 (1.2) | 0 | 0 | 0 | 0 | 0 | 0 |  |
| Hepatic failure | 0 | 0 | 1 (1.2) | 0 | 0 | 0 | 0 | 0 | 0 | 0 |  |
| Hyperbilirubinemia | 0 | 1 (1.2) | 0 | 0 | 0 | 0 | 0 | 0 | 0 | 0 |  |

One hepatic TEAE with missing severity was not included in this analysis. TEAEs are listed as reported by study sites.
^a^ INBRX-105–related TEAEs were those with a relationship of possible, probable, or very likely/certainly related to INBRX-105 as determined by the investigator. For a given preferred term, a patient was counted only once, even if multiple events for that preferred term were reported. The following preferred terms were considered hepatic TEAEs: acute hepatic failure, alanine aminotransferase decreased, alanine aminotransferase increased, aspartate aminotransferase increased, bile duct obstruction, bile duct stenosis, bile duct stone, biliary dilatation, biliary fistula, biliary obstruction, blood alkaline phosphatase, blood alkaline phosphatase increased, blood bilirubin increased, cholangitis, cholecystitis, cholelithiasis, cholestatic pruritus, drug-induced liver injury, feces discolored, gallbladder obstruction, gamma-glutamyltransferase increased, hepatic encephalopathy, hepatic enzyme increased, hepatic failure, hepatic function abnormal, hepatitis acute, hepatorenal syndrome, hyperbilirubinemia, hypertransaminasemia, immune-mediated hepatitis, ischemic hepatitis, jaundice, liver abscess, liver disorder, liver function test increased, liver injury, and transaminases increased.

Abbreviations: ALP, alkaline phosphatase; ALT, alanine aminotransferase; AST, aspartate aminotransferase; TEAE, treatment-emergent adverse event.
