# Supplementary material for: Phase 1 Study of INBRX-105, a TNFRSF9 (4-1BB) and PD-L1 Bispecific Antibody, in Patients with Select Solid Tumors
Source: Cancer Res Commun. 2026 Feb 23;6(2):374–82. doi: 10.1158/2767-9764.CRC-25-0577 (PMC13143200; doi:10.1158/2767-9764.CRC-25-0577)
Supplement: Table S5 — summarizes the treatment-emergent adverse events that resulted in death [file crc-25-0577_table_s5_suppst5.docx]

**Supplementary Table S5. TEAEs resulting in death**

| **Preferred term** | **INBRX-105 (n=81), n (%)** | **INBRX-105 + pembrolizumab**  **(n=79), n (%)** |
| --- | --- | --- |
| **Patients with TEAE resulting in death** | 3 (3.7) | 6 (7.6)^a^ |
| Multiple organ dysfunction syndrome | 1 (1.2) | 1 (1.3) |
| Tumor hemorrhage | 1 (1.2) | 0 |
| Urosepsis | 1 (1.2) | 1 (1.3) |
| Sepsis | 0 | 2 (2.5) |
| Coronavirus infection | 0 | 1 (1.3) |
| Cytokine release syndrome | 0 | 1 (1.3) |
| Respiratory failure | 0 | 1 (1.3) |
| ^a^ One patient who received combination therapy experienced 2 TEAEs that were each reported as having a fatal outcome (sepsis and multiple organ dysfunction syndrome).  Abbreviation: TEAE, treatment-emergent adverse event. | | |
