# Supplementary material for: Phase 1 Study of INBRX-105, a TNFRSF9 (4-1BB) and PD-L1 Bispecific Antibody, in Patients with Select Solid Tumors
Source: Cancer Res Commun. 2026 Feb 23;6(2):374–82. doi: 10.1158/2767-9764.CRC-25-0577 (PMC13143200; doi:10.1158/2767-9764.CRC-25-0577)
Supplement: Table S6 — shows the pharmacokinetic parameter estimates for INBRX-105 in cycle 1 [file crc-25-0577_table_s6_suppst6.docx]

**Supplementary Table S6. Pharmacokinetic parameter estimates for INBRX-105 for cycle 1**

| **Dose, mg/kg  [patients, n]** | **C_max_, mean (SD), ng/mL [patients, n]** | **AUC_inf_, mean (SD), ng*day/mL [patients, n]** | **t_1/2,z_, mean (SD), days [patients, n]** |
| --- | --- | --- | --- |
| 0.001 [1] | 4.5 [1] | NE | NE |
| 0.003 [7] | 77.4 (99.9) [7] | 133 [2] | 2.5 [2] |
| 0.01 [3] | 200 (217) [3] | 274 [2] | 4.1 [2] |
| 0.03 [8]^a^ | 482 (174) [8] | 389 (270) [3] | 0.3 (1.5) [3] |
| 0.1 [13]^a^ | 1740 (631) [13] | 2488 (793) [8] | 1.5 (2.3) [8] |
| 0.3 [97]^a^ | 5624 (3238) [97] | 12,160 (4567) [85] | 1.2 (0.4) [85] |
| 1.0 [21]^a^ | 18,700 (7041) [21] | 38,657 (16,895) [15] | 1.7 (1.0) [15] |
| 3.0 [3] | 70,500 (36100) [3] | 156,000 (67,900) [3] | 3.8 (0.5) [3] |
| Doses were administered every 2 weeks. ^a^ Includes patients in the monotherapy (parts 1 and 2) and combination (parts 3 and 4) cohorts. Abbreviations: AUC_inf_, area under the concentration-time curve from time 0 extrapolated to infinity; C_max_, maximum serum concentration; NE, not evaluable (ie, insufficient data to estimate parameter); t_½,z_, terminal elimination half-life associated with the terminal slope. | | | |
