# Supplementary material for: Phase 1 Study of INBRX-105, a TNFRSF9 (4-1BB) and PD-L1 Bispecific Antibody, in Patients with Select Solid Tumors
Source: Cancer Res Commun. 2026 Feb 23;6(2):374–82. doi: 10.1158/2767-9764.CRC-25-0577 (PMC13143200; doi:10.1158/2767-9764.CRC-25-0577)
Supplement: Table S7 — summarizes the characteristics of patients who were responders to study treatment [file crc-25-0577_table_s7_suppst7.docx]

**Supplementary Table S7. Responder characteristics.**

| **Study part** | **INBRX-105 dose (mg/kg)** | **CPS/TPS** | **CPI status** | **Best response** | **Tumor type** |
| --- | --- | --- | --- | --- | --- |
| **Single-agent dose expansion** | 0.3 | 25 | R/R | PR | Head and neck |
|  | 0.3 | 90 | R/R | CR | Head and neck |
|  | 1 | 90 | R/R | PR | NSCLC |
| **Combination dose escalation** | 0.03 | Missing | Naive | PR | Head and neck |
|  | 1 | 5 | R/R | PR | Head and neck |
|  | 0.3 | Missing | Naive | PR | Head and neck |
|  | 0.3 | 10 | R/R | PR | Gastric |
| **Combination dose expansion** | 0.3 | 5 | Naive | PR | Head and neck |
|  | 0.3 | 2 | Naive | PR | NSCLC |
|  | 0.3 | 25 | R/R | PR | Melanoma |
|  | 0.3 | 5 | R/R | PR | Melanoma |
|  | 0.3 | 100 | Naive | CR | Head and neck |
|  | 0.3 | 2 | Naive | PR | Head and neck |
|  | 0.3 | Missing | R/R | PR | Melanoma |

CPI, checkpoint inhibitor; CPS, combined positive score; CR, complete response; NSCLC, non-small cell lung cancer; PR, partial response; R/R, relapsed/refractory; TPS, tumor proportion score.
